# Supplementary material for: Nasopharyngeal carcinoma cells promote regulatory T cell development and suppressive activity via CD70-CD27 interaction
Source: Nat Commun. 2023 Apr 6;14:1912. doi: 10.1038/s41467-023-37614-6 (PMC10079957; doi:10.1038/s41467-023-37614-6)
Supplement: Supplementary file 1 — Supplementary Information [file 41467_2023_37614_MOESM1_ESM.pdf]

Supplementary Information

Nasopharyngeal carcinoma cells promote regulatory T cell development and suppressive activity via CD70-CD27 interaction

Lanqi Gong[1,2*], Jie Luo[1*], Yu Zhang[3,4,5*], Yuma Yang[1], Shanshan Li[2], Xiaona Fang[1], Baifeng Zhang[1], Jiao Huang[1], Larry Ka-Yue Chow[1], Dittman Chung[1], Jinlin Huang[1], Cuicui Huang[1,2], Qin Liu[1,2], Lu Bai[1,2], Yuen Chak Tiu[1], Pingan Wu[6], Yan Wang[7], George Sai-Wah Tsao[8], Dora Lai-wan Kwong[1,2], Anne Wing-Mui Lee[1,2,9], Wei Dai[1,2], Xin-Yuan Guan[1,2,4,5,9#]

[1]Department of Clinical Oncology, Li Ka Shing Faculty of Medicine, The University of Hong Kong, Hong Kong, China. [2]Department of Clinical Oncology, The University of Hong Kong-Shenzhen Hospital, Shenzhen, China. [3]Department of Pediatric Oncology, Sun Yat-sen University Cancer Center, Guangzhou, China. [4]State Key Laboratory of Oncology in South China, Guangdong Key Laboratory of Nasopharyngeal Carcinoma Diagnosis and Therapy, Sun Yat-sen University Cancer Center, Guangzhou, China. [5]Collaborative Innovation Center for Cancer Medicine, Sun Yat-sen University Cancer Center, Guangzhou, China. [6]Department of Surgery, The University of Hong Kong-Shenzhen Hospital, Shenzhen, China. [7]Department of Pathology, The University of Hong Kong-Shenzhen Hospital, Shenzhen, China. [8]School of Biomedical Sciences, Li Ka Shing Faculty of Medicine, The University of Hong Kong, Hong Kong, China. [9]Advanced Energy Science and Technology Guangdong Laboratory, Huizhou, China.

*These authors contributed equally

#Correspondence: Xin-Yuan Guan, Department of Clinical Oncology, Li Ka Shing Faculty of Medicine, The University of Hong Kong, Laboratory Block L10-56, 21 Sassoon Road, Hong Kong, China. Tel: 852-3917-9782; E-mail: xyguan@hku.hk

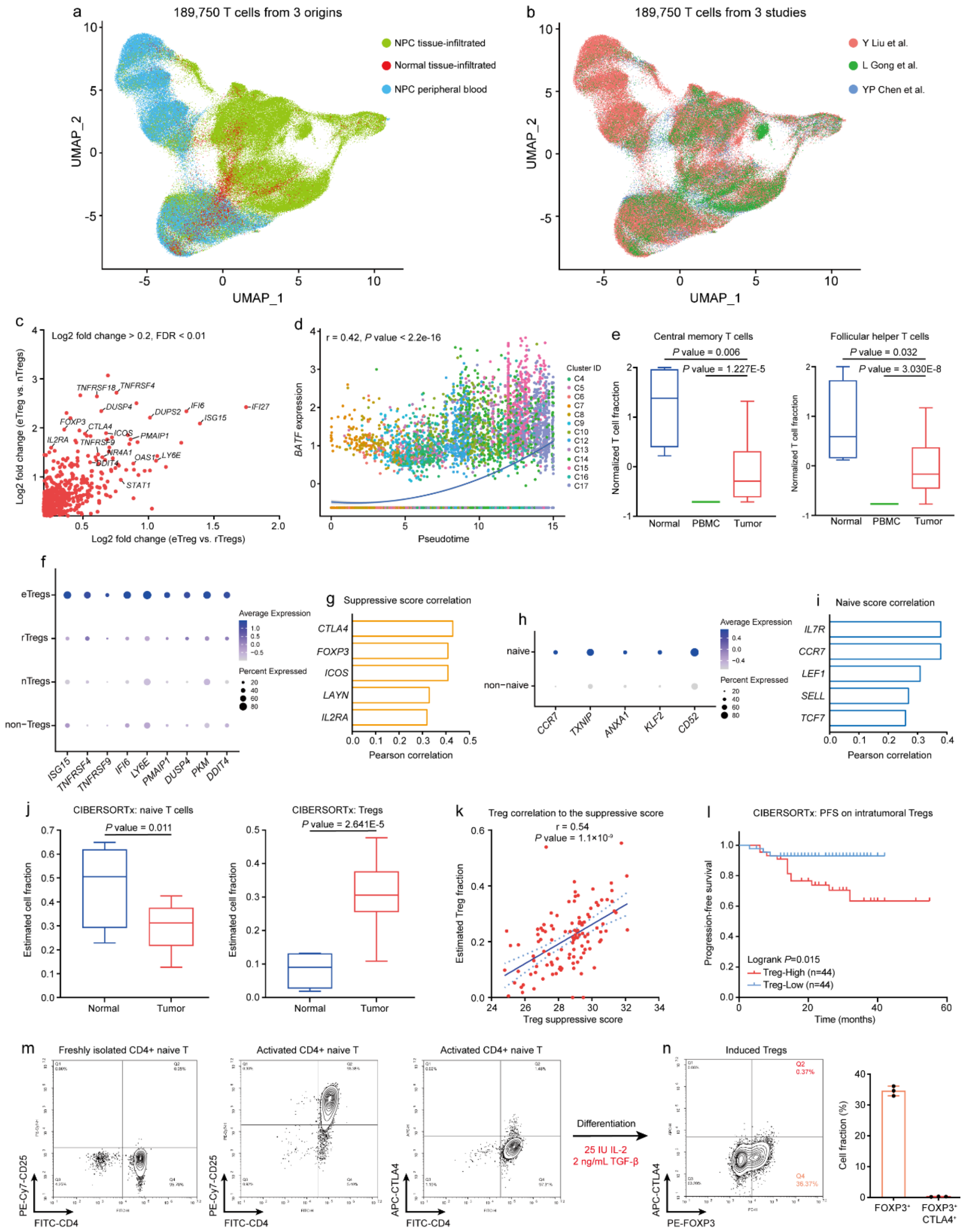

Supplementary Figure 1 The NPC microenvironment harbors an enriched and activated Treg population.

a The UMAP plot of 189,750 T cells from 3 sample origins. **b** The UMAP plot of 189,750 T cells from 3 single-cell NPC cohorts. **c** The differentially expressed genes between eTregs and nTregs/rTregs, determined by the MAST analysis. **d** The change of *BATF* expression through the pseudotime developmental process from CD4+ naïve T cells to T_{FH} cells, modeled by two-sided polynomial regression analysis with the 95% confidence band. **e** The normalized fractions of CD4+ central memory T cells and

CD4+ follicular helper T cells in NPC tissues (n=36) versus in NPC peripheral blood (n=10) and INP tissues (n=4) (two-sided unpaired t test). **f** The 9-gene signature used to construct Treg suppressive module and significantly enriched in the eTreg population. **g** The Pearson correlation (two-sided) between the Treg suppressive score and Treg-specific signatures in the NPC scRNA-seq cohort. **h** The 5-gene signature used to construct T cell naiveness module and significantly associated with naïve T cells. **i** The Pearson correlation (two-sided) between the T cell naïve score and naïve signatures in the NPC scRNA-seq cohort. **j** The estimated fraction of naïve T cells and Tregs by CIBERSORTx deconvolution of NPC bulk RNA-seq cohort GSE68799 (tumor n=42 and normal n=3, two-sided unpaired t test). **k** Pearson correlation (two-sided) between the Treg faction estimated by CIBERSORTx and Treg suppressive score computed by the Treg linear model (GSE102349, n=112). **l** The progression-free survival for NPC patients from GSE102349, stratified by the estimated Treg fraction (high n=44, low n =44, two-sided log-rank test). **m** Immunophenotyping of freshly isolated (left panel) and TCR-activated (middle and right panels) CD4+ naïve T cells by CD4, CD25 and CTLA4 staining. **n** Left, immunophenotyping of CD4+ naïve T cells after 3-day induced Treg differentiation without tumor cell co-culture. Right, the fraction of FOXP3+ induced Tregs and FOXP3+/CTLA4+ activated Tregs (n=3). The n number represents n biologically independent samples/experiments in each group. The data are presented as the mean ± SD (bar plots), median ± IQR (whiskers = 1.5×IQR, box & whiskers plots) and KDE (violin plots).

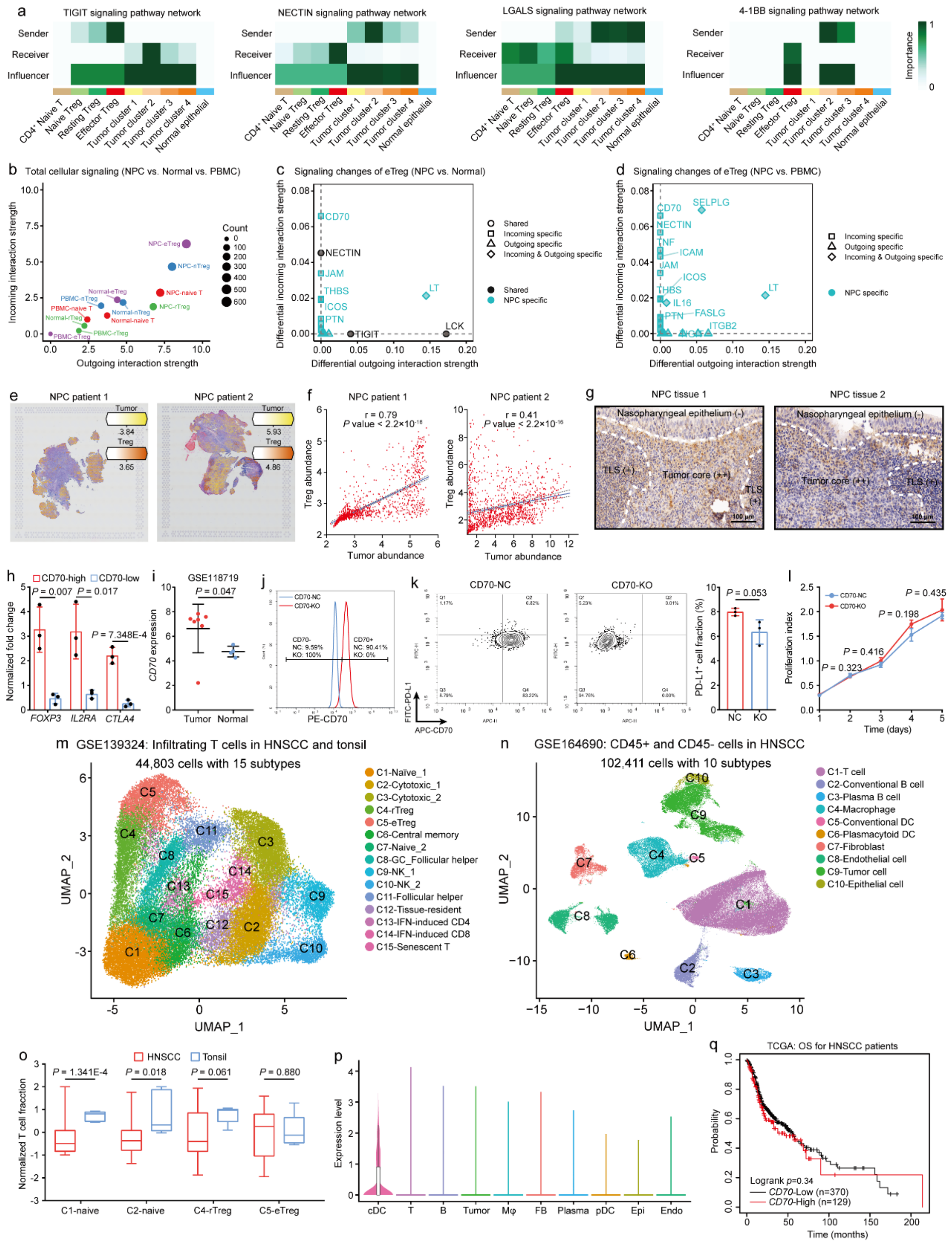

Supplementary Figure 2 CD70-CD27 interaction plays an NPC-specific role in Treg regulation.

a Other enriched cellular communication between NPC and CD4+ naïve T cells/Tregs. **b** Total cellular signaling strength in CD4+ T cells and Treg subtypes from the NPC, INP and NPC peripheral blood samples. **c, d** The differentially expressed cell-cell communications between NPC-infiltrated eTregs and INP-infiltrated eTregs **(c)** and peripheral eTregs (**d**). **e** Spatial co-localization of NPC cells and Tregs in primary NPC

tissues, estimated by cell2location. **f** Spearman correlation (two-sided) between NPC cells and Tregs. **g** IHC staining revealed CD70 expression in the tertiary lymphoid structure, tumor core, and normal epithelium. **h** The 18s-normalized mRNA fold changes of Treg markers in CD70-high and CD70-low NPC patients (n=3, two-sided unpaired t test). **i** Normalized CD70 expression in GSE118719. (tumor n=7, normal n=3) (two-sided unpaired t test). **j** The fraction of CD70+ cells after CD70-KO in C666 cells. **k** The fraction of CD70+ and PD-L1+ cells in CD70-NC and CD70-KO C666 cells (n=3, two-sided unpaired t test). **l** The in vitro proliferation of CD70-NC and CD70-KO C666 cells (n=3, two-sided unpaired t test). **m** The UMAP plot of 44,803 T cells with 15 subtypes identified from HNSCC and normal tonsil scRNA-seq samples (GSE139324). **n** The UMAP plot of 102,411 cells with 10 major cell lineages identified from CD45+ and CD45- HNSCC samples (GSE164690). **o** The normalized fractions of C1-naïve T cells, C2-naïve T cells, C4-rTregs and C5-eTregs in HNSCC tissues (n=26) and normal tonsil tissues (n=5) from GSE139324 (two-sided unpaired t test). **p** Single-cell expression of CD70 across major cell lineages in GSE164690. **q** The progression-free survival for HNSCC patients from TCGA cohort, stratified by the CD70 expression (high n=129, low n=370, two-sided log-rank test). The n number represents n biologically independent samples/experiments in each group. The data are presented as the mean ± SD (bar plots), median ± IQR (whiskers = 1.5×IQR, box & whiskers plots) and KDE (violin plots).

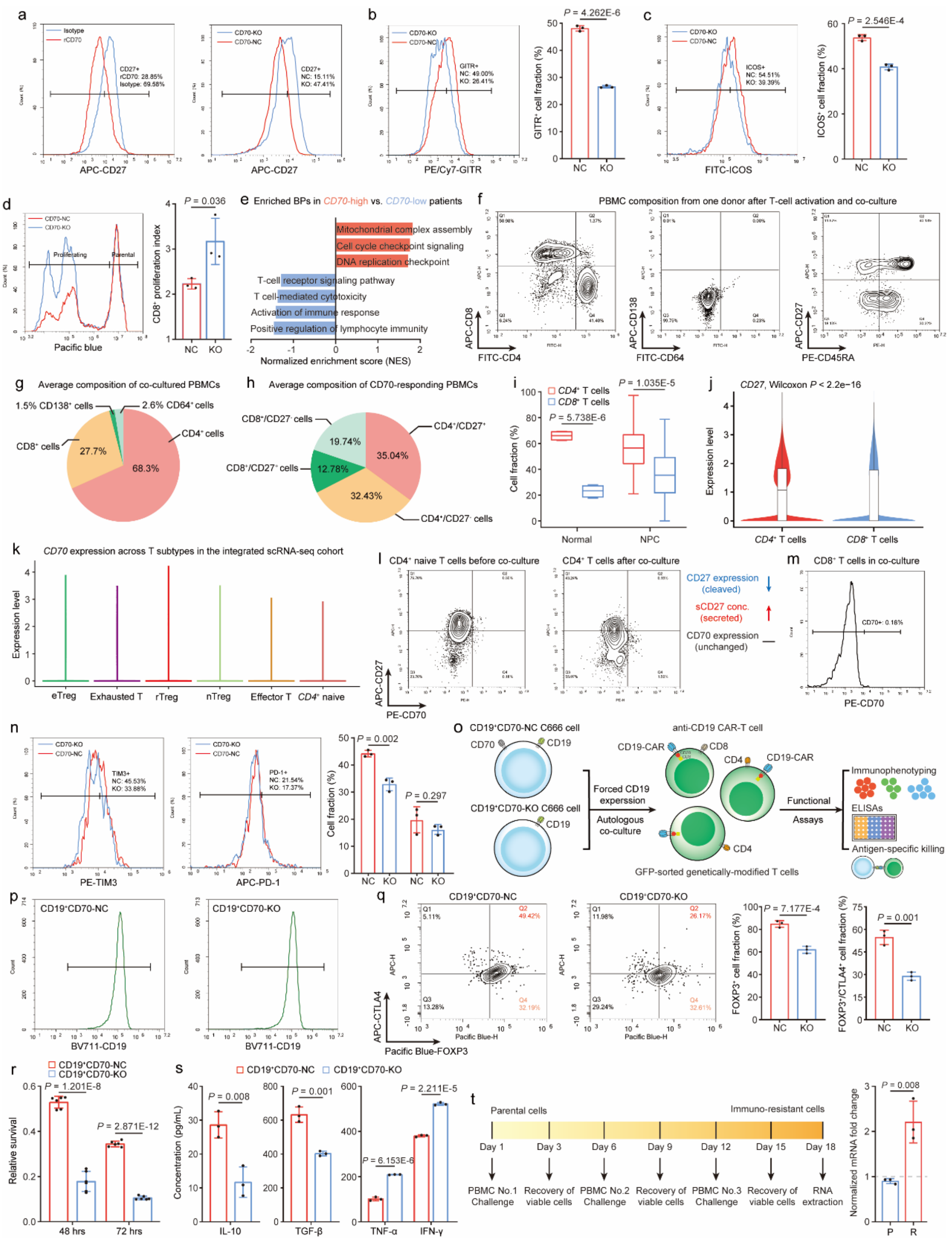

Supplementary Figure 3 CD70 knockout promotes T-cell cytotoxicity by inhibiting Treg development and activation.

a Flow analysis of CD27 expression in IgG/rCD70-treated and co-cultured CD4+ naïve T cells. **b, c** The fraction of GITR+ (**b**) and ICOS+ (**c**) cells in the co-cultured CD4+ naïve T cells (n=3, two-sided unpaired t test). **d** The suppressive activity of co-cultured CD4+ naïve T cells on paired CD8+ T cells (n=3, two-sided unpaired t test). **e** The enriched pathways in CD70-high/low NPC patients in GSE102349 (n=112). **f** Flow analysis of CD4, CD8, CD64, CD138, CD45RA and CD27 expression in co-cultured

PBMCs. **g** The fraction of CD4+, CD8+ T cells, CD64+ monocytes and CD138+ B cells in co-cultured PBMCs (n=3). **h** The fraction of T subtypes in co-cultured PBMCs (n=3). **i** The fractions of CD4+/CD8+ T cells in NPC (n=36) and INP (n=4) samples (two-sided unpaired t test). **j** The single-cell expression of *CD27* in CD4+ and CD8+ T cells (n=36, two-sided Wilcoxon signed-rank test). **k** The single-cell expression of *CD70* in T subtypes. **l, m** Flow analysis of CD70/CD27 expression in CD4+ (**l**) and CD70 expression in CD8+ T cells (**m**) in NPC co-culture systems. **n** The fractions of TIM3+/PD-1+ in CD8+ T cells in PBMC co-culture systems (n=3, two-sided unpaired t test). **o** Illustration of the antigen-specific co-culture between CD19+CD70-NC/CD19+CD70-KO C666 cells and anti-CD19 CAR-T cells. **p** Flow analysis of CD19 overexpression in CD70-NC and CD70-KO cells. **q** The fractions of FOXP3+ and FOXP3+/CTLA4+ Tregs in the antigen-specific co-culture systems (n=3, two-sided unpaired t test). **r** Antigen-specific T-cell cytotoxicity in the PBMC co-culture systems (n=3, two-sided unpaired t test). **s** The change of immunosuppressive and cytotoxic cytokines in antigen-specific NPC/CD4+ naïve T cell and PBMC co-culture systems (n=3, two-sided unpaired t test). **t** The mRNA change of *CD70* between parental and immuno-resilient C666 cells (n=3, two-sided unpaired t test). The n number represents n biologically independent samples/experiments in each group. The data are presented as the mean ± SD (bar plots), median ± IQR (whiskers = 1.5×IQR, box & whiskers plots) and KDE (violin plots).

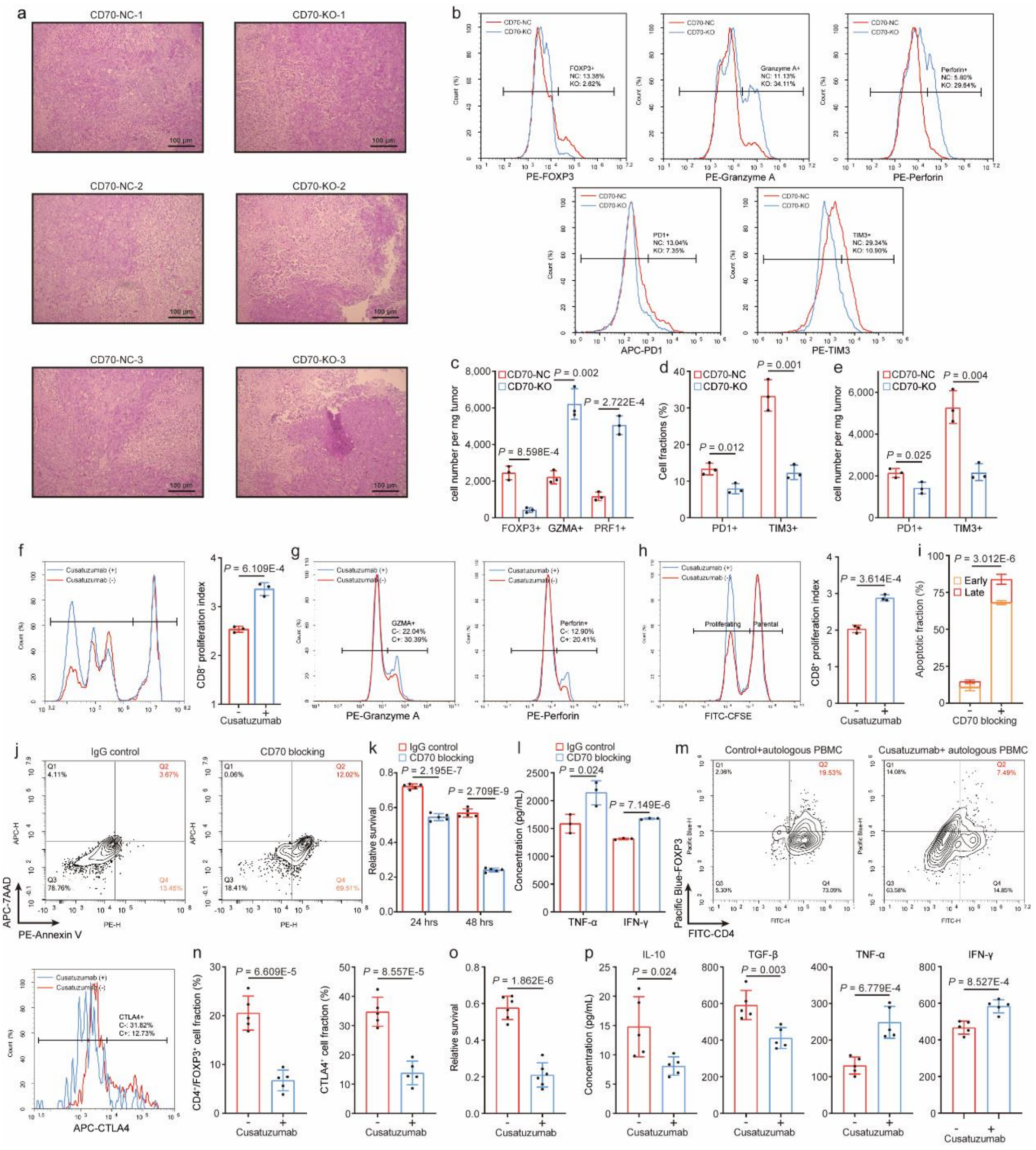

Supplementary Figure 4 Therapeutic CD70 blocking reverts Treg-mediated immunosuppression.

a The representative H&E staining images showed immune infiltration in CD70-NC and CD70-KO tumors from humanized mice (n=3). **b** Flow analysis of FOXP3, Granzyme A and perforin expression in infiltrating CD4+ T cells and CD8+ T cells from CD70-NC and CD70-KO tumors. **c** Total numbers of FOXP3+, Granzyme A+ and perforin+ cells per mg of CD70-NC and CD70-KO (n=3, two-sided unpaired t test). **d** Fractions of TIM3+ and PD-1+ cells amongst CD8+ T cells from CD70-NC and CD70-KO tumors (n=3, two-sided unpaired t test). **e** Total numbers of TIM3+ and PD-1+ cells per mg of CD70-NC and CD70-KO tumors (n=3, two-sided unpaired t test). **f** The suppressive activity of co-cultured CD4+ naïve T cells on paired CD8+ T cells (n=3, two-sided unpaired t test). **g** Flow analysis of Granzyme A and perforin

expression in CD8+ T cells in the PBMC co-culture systems. **h** The change of CD8+ T-cell proliferation in the PBMC co-culture systems. **i-k** T-cell cytotoxicity measured in the IgG-treated and CD70 blocking antibody-treated C666 (**i** and **j**, flow cytometry, n=3; **k**, XTT assay, n=5) PBMC co-culture systems (two-sided unpaired t test). **l** The change of CD8+ T-cell cytotoxic cytokines in the PBMC co-culture systems (n=3, two-sided unpaired t test). **m**, **n** The fractions of CD4+/FOXP3+ and CD4+/FOXP3+/CTLA4+ Tregs amongst autologous PBMCs co-cultured with IgG-treated and cusatuzumab-treated primary NPC cells (n=5, two-sided unpaired t test). **o** T-cell cytotoxicity measured in the autologous PBMC co-culture system (n=6, two-sided unpaired t test). **p** The change of T-cell immunosuppressive and cytotoxic cytokines in the autologous PBMC co-culture system (n=5, two-sided unpaired t test). The n number represents n biologically independent samples/experiments in each group. The data are presented as the mean ± SD (bar plots).

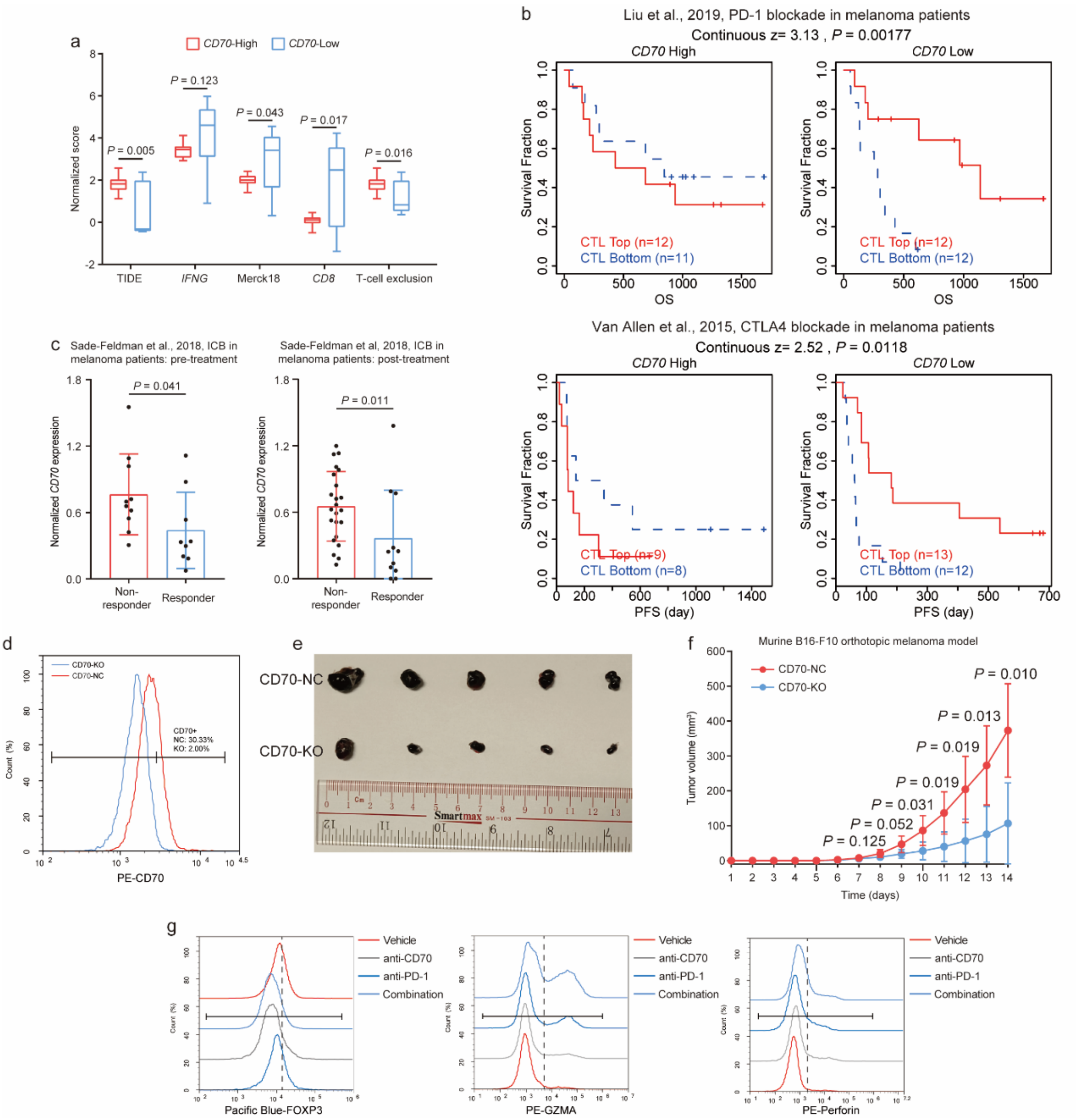

Supplementary Figure 5 ***CD70*** **expression correlates to immunotherapy responses in NPC and melanoma patients.**

a ICB responsiveness (TIDE score), T-cell dysfunction, and exclusion scores in *CD70*-high and *CD70*-low NPC patients with top 10 highest TIDE scores (n=10, two-sided unpaired t test), predicted by the TIDE module (http://tide.dfci.harvard.edu/). **b** The correlation between *CD70* and T cell dysfunction and inferior treatment outcome in melanoma patients received with PD-1 blockade (n=47) in the Liu et al. cohort[1] and melanoma patients received with CTLA4 blockade (n=42) in the Van Allen et al. cohort[2] (two-sided log-rank test). **c** The correlation between *CD70* and ICB responses in pre (non-responder n=10, responder n=9) and post-treatment (non-responder n=23, responder n=11) melanoma patients in the Sade-Feldman et al. cohort[3] (two-sided unpaired t test). **d** Flow analysis of CD70 expression in B16-F10 melanoma cells after CRISPR-mediated CD70-KO. **e** The representative image of freshly dissected CD70-NC and CD70-KO B16-F10 tumors in C57BL/6 mice (n=5). **f** The CD70-NC and

CD70-KO B16-F10 tumor growth in C57BL/6 mice (n=5, two-sided unpaired t test). **g** Flow analysis of FOXP3, Granzyme A and perforin expression in CD4+ T cells and CD8+ T cells isolated from IgG-treated, cusatuzumab-treated, camrelizumab-treated and combination-treated Xeno76 tumors in humanized mice. The n number represents n biologically independent samples/experiments in each group. The data are presented as the mean ± SD (bar plots) and median ± IQR (whiskers = 1.5×IQR, box & whiskers plots).

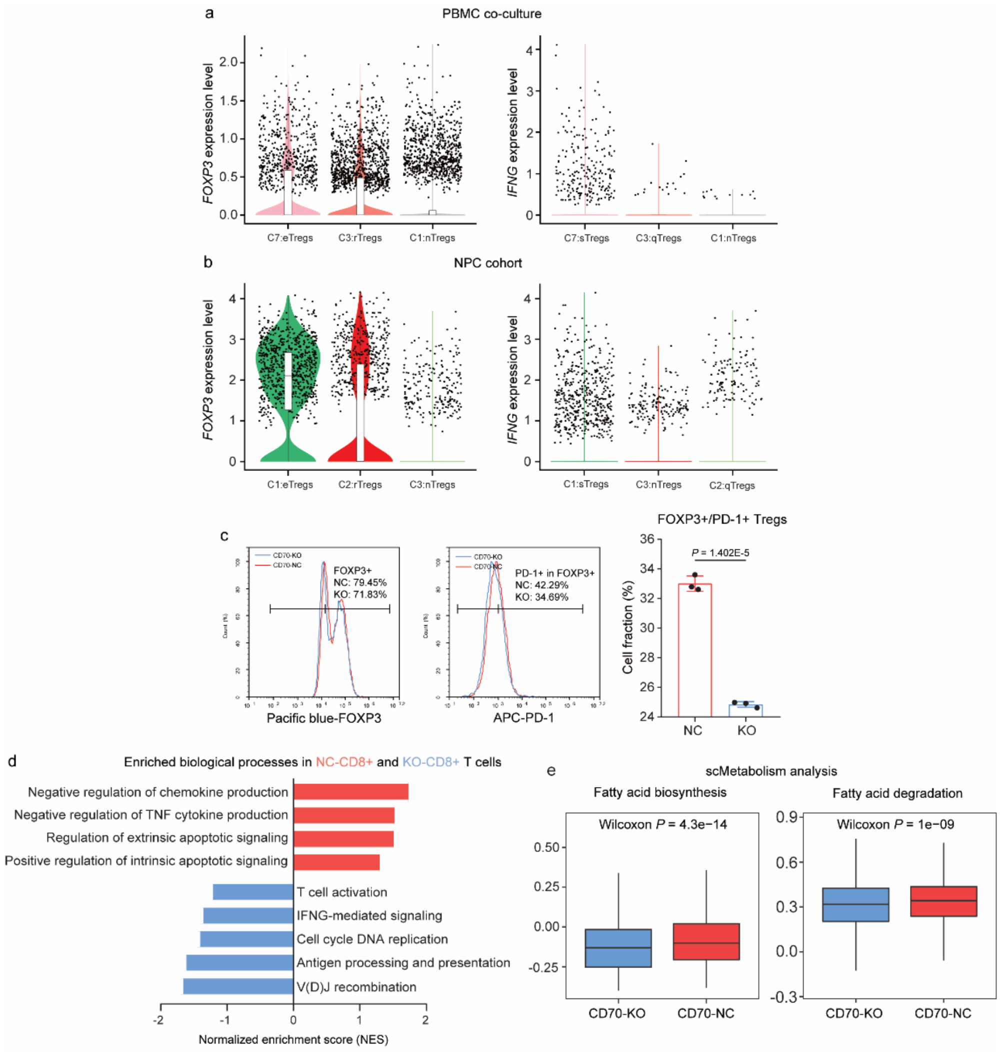

Supplementary Figure 6 Single-cell analysis reveals CD70-mediated immunophenotyping alterations in Tregs and CD8+ T cells.

a The expression level of FOXP3 and IFNG in nTregs, rTregs, and eTregs in the co-cultured PBMC single-cell cohort (n=3168, 2446, and 1381). **b** Expression level of FOXP3 and IFNG in nTregs, rTregs, and eTregs in the integrated NPC single-cell cohort (n=4756, 6992 and 8935). **c** PD-1 expression on FOXP3+ Tregs co-cultured with CD70-NC and CD70-KO NPC cells. Left: flow cytometry analysis of expression of surface PD-1 in co-cultured FOXP3+ Tregs; Right: the quantified fraction of PD-1+/FOXP3+ in the PBMC co-culture system with CD70-NC and CD70-KO C666 cells (n=3, two-sided unpaired t test). **d** Enriched GO:BP pathways associated with immune response in NC-CD8+ T cells and KO-CD8+ T cells, revealed by GSEA. **e** Enriched fatty acid metabolism-associated activities in NC-Tregs and KO-Tregs, identified by scMetabolism analysis (n=3731 and 3264, two-sided Wilcoxon signed-rank test). The n number represents n biologically independent cells/samples/experiments in each group. The data are presented as the mean ± SD (bar plots), median ± IQR (whiskers = 1.5×IQR, box & whiskers plots) and KDE (violin plots).

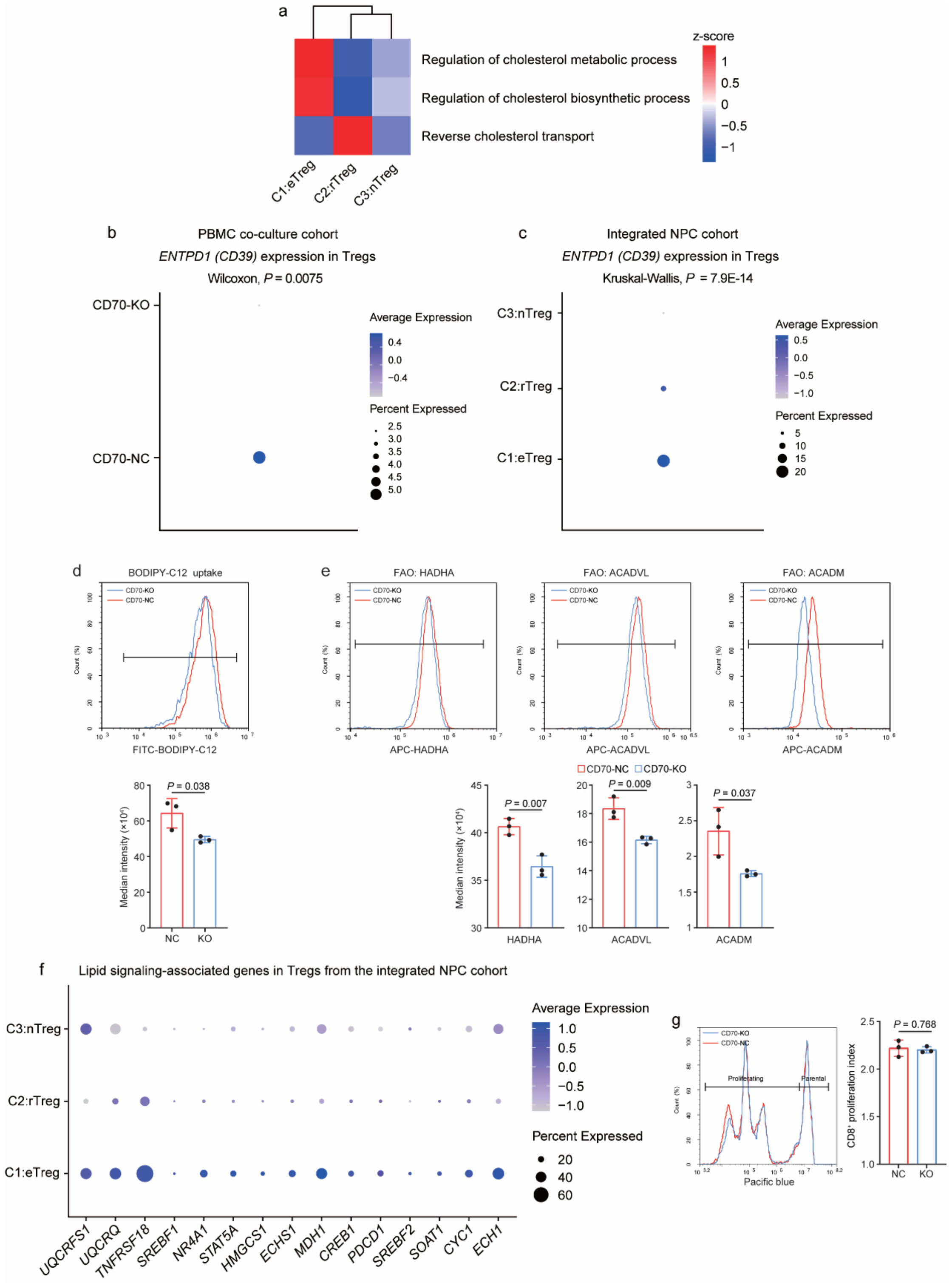

Supplementary Figure 7 CD70+ NPC cells influence lipid signaling in co-cultured CD4+ naïve T cells and Tregs.

a GSVA revealed cholesterol-associated activities in Treg subtypes from the integrated NPC single-cell cohort. **b** Expression of *ENTP1* (encodes CD39) in NC-Tregs and KO-

Tregs in the PBMC co-culture cohort (two-sided Wilcoxon signed-rank test). **c** Expression of *ENTP1* across three Treg subtypes in the integrated NPC cohort (Kruskal-Wallis one-way analysis of variance). **d** The median intensity of intracellular BODIPY-C12 taken up by Tregs co-cultured with CD70-NC and CD70-KO C666 cells (n=3, two-sided unpaired t test). **e** The median intensity of three key FAO enzymes in Tregs co-cultured with CD70-NC and CD70-KO C666 cells (n=3, two-sided unpaired t test). **f** The single-cell expression and average of 15 representative gene associated with lipid signaling in eTregs, rTregs and nTregs from the NPC scRNA-seq cohort (Wilcoxon signed-rank test). **g** The suppressive activity of co-cultured CD4+ naïve T cells without lipid supplementation on paired CD8+ T cells (n=3, two-sided unpaired t test). The n number represents n biologically independent samples/experiments in each group. The data are presented as the mean ± SD (bar plots).

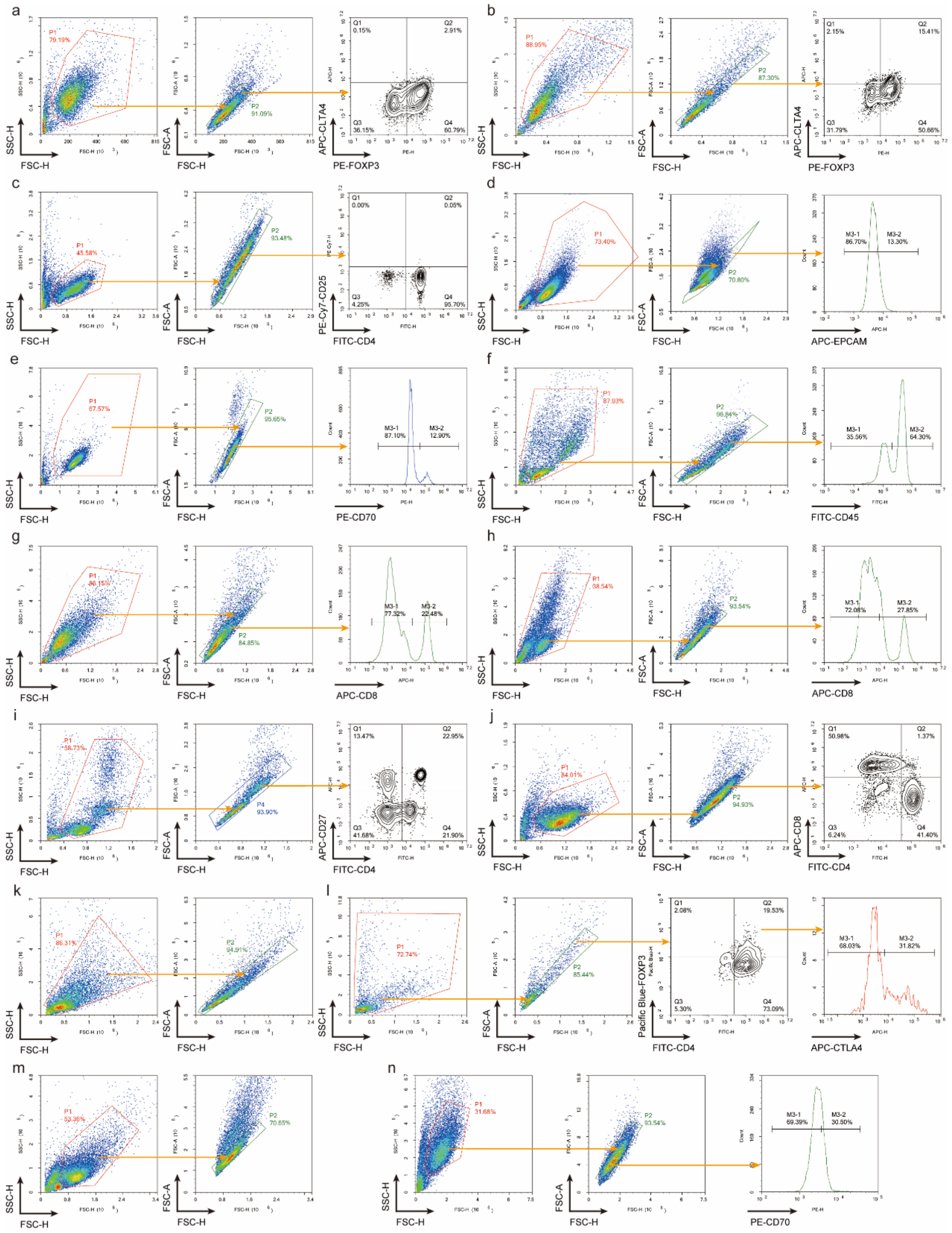

Supplementary Figure 8 Gating strategy for flow cytometry analysis.

a Gating strategy for flow cytometry analysis of direct co-culture assays between CD4+ naïve T cells/Tregs and NPC/NPE cells, including Figures 1j, 2n, 3a, 3c, 3e, 3g, 4a, 4c, 6c, 6g, 6h, 6p, 6q, 6v, 6w, and Supplementary Figures 3a-3c, 3i, 3m, 3q, 6c, 7d and 7e.

b Gating strategy for flow cytometry analysis of transwell-based co-culture assays between CD4+ naïve T cells and NPC/NPE cells, including Figure 1j.

c Gating strategy for flow cytometry analysis of CD4+ naïve T differentiation assay without NPC/NPE cell co-culture, including Supplementary Figures 1m and 1n.

d Gating strategy for flow cytometry analysis of primary NPC tissues, including Figure 2e.

e Gating strategy for flow cytometry analysis of NPC/NPE cell lines, including Figures 2m, 7a, 7i, 7j and Supplementary Figures 2j, 2k and 2p, as well as flow sorting of CD70- C666 and B16-F10 cells.

f Gating strategy for flow cytometry analysis of NPC/PBMC killing assays, including Figures 3j, 3k, 4e, 4f, 4i, 4n and Supplementary Figures 4i and 4j.

g Gating strategy for flow cytometry analysis of CD8+ T cells in co-cultured PBMCs, including Figures 3m, 3n, 4h, and Supplementary Figures 3n and 4g.

h Gating strategy for flow cytometry analysis of CD8+ T cell proliferation in co-cultured PBMCs and Treg suppression assays, including Figures 3p, 3q and Supplementary Figures 3d, 4f, 4h and 7g.

i Gating strategy for flow cytometry analysis of isolated PBMCs, including Supplementary Figures 3f and 3h.

j Gating strategy for flow cytometry analysis of expanded PBMCs, including Supplementary Figures 3f and 3g.

k Gating strategy for flow cytometry analysis of tumor-infiltrating T cells from humanized mice, including Figures 3t, 4q and Supplementary Figures 4b-4e and 5g.

l Gating strategy for flow cytometry analysis of co-culture assays using primary NPC cells, including Supplementary Figures 4m-4n.

m Gating strategy for flow cytometry analysis of Xeno76, including Figure 4j.

n Gating strategy for flow cytometry analysis of the B16-F10 cell line, including Supplementary Figure 5d.

Supplementary Table 1 Clinical parameter associated with Treg suppressive score in 42 NPC patients from GSE68799

Characteristics	Treg suppressive score		Statistics	
Stage	Low	High	Chi-square	*P* value
II	4 (66.7%)	2 (33.3%)		
III	16 (44.4%)	20 (55.6%)	1.018	0.400
Age				
>60	14 (48.3%)	15 (51.7%)		
<60	6 (46.2%)	7 (53.8%)	0.016	1.000
Sex				
Female	4 (44.4%)	5 (55.6%)		
Male	16 (48.5%)	17 (51.5%)	-0.033	1.000

*Two-sided chi-square test was used to compute the statistical significance.

Supplementary Table 2 The mass spectrometry result of intracellular fatty acids in CD4+ naïve T cells co-cultured with CD70-NC and CD70-KO C666 cells.

nmol/million cells	CD70-NC	CD70-KO
C4:0	0	0
C6:0	0	0
C8:0	0	0
C10:0	0	0
C11:0	0	0
C12:0	0.231629	0.205992
C13:0	0	0
C14:0	1.068738	0.866816
C14:1	0	0
C15:0	0.144098	0.118907
C15:1	0.143844	0.123486
C16:0	7.317763	5.880865
C16:1	0.461141	0.311316
C17:0	0.240546	0.190259
C17:1	0	0
C18:0	5.173723	4.222482
C18:1, trans	0	0
C18:1, cis	4.117237	3.000295
C18:2, trans	0	0
C18:2, cis	1.512408	1.131018
C18:3 n6	0	0
C18:3 n3	0	0
C20:0	1.509817	1.304967
C20:1 n9	0.177313	0.125406
C20:2	0.282357	0.193916
C21:0	0.01739	0.013381
C20:3 n6	0.703868	0.403206
C20:4 n6	4.36168	3.332361
C20:3 n3	0	0
C20:5	0.475872	0.419609
C22:0	1.846567	1.605485
C22:1	0	0
C22:2	0	0
C23:0	0	0
C24:0	2.06315	1.763007
C22:6	3.776627	2.643538
C24:1	0.270887	0.214843
C26:0	1.976447	1.721675
Total	37.8731	29.79283

Supplementary Table 3 qRT-PCR primers used in this study.

qRT-PCR primers	
Gene	Sequencing (5' - 3')
*FOXP3*_forward	GTGGCCCGGATGTGAGAAG
*FOXP3*_reverse	GGAGCCCTTGTCGGATGATG
*IL2RA*_forward	CGCAGAATAAAAAGCGGGTCA
*IL2RA*_reverse	ACTTGTTTCGTTGTGTTCCGA
*IKZF2*_forward	GCAGCCTAGAAGAACCCCTAA
*IKZF2*_reverse	CATTCGGAAGCCGGATTCCT
*CTLA4*_forward	GCCCTGCACTCTCCTGTTTTT
*CTLA4*_reverse	GGTTGCCGCACAGACTTCA
*LAYN*_forward	ACAGAGCTGACAACACCTGTA
*LAYN*_reverse	GATGTAGGCCAGATTCAAGGC
*SOX4*_forward	GACCTGCTCGACCTGAACC
*SOX4*_reverse	CCGGGCTCGAAGTTAAAATCC
*TNFRSF4*_forward	GCAATAGCTCGGACGCAATCT
*TNFRSF4*_reverse	GAGGGTCCCTGTGAGGTTCT
*TNFRSF9*_forward	AGCTGTTACAACATAGTAGCCAC
*TNFRSF9*_reverse	GGACAGGGACTGCAAATCTGAT
*ICOS*_forward	ACAACTTGGACCATTCTCATGC
*ICOS*_reverse	TGCACATCCTATGGGTAACCA
*IL10*_forward	GACTTTAAGGGTTACCTGGGTTG
*IL10*_reverse	TCACATGCGCCTTGATGTCTG
*TGFB1*_forward	CAATTCCTGGCGATACCTCAG
*TGFB1*_reverse	GCACAACTCCGGTGACATCAA
*MKI67*_forward	GCCTGCTCGACCCTACAGA
*MKI67*_reverse	GCTTGTCAACTGCGGTTGC
*TNFRSF18*_forward	CAGTCCCAGGGGAAATTCAGT
*TNFRSF18*_reverse	GAACACAGTGAGAAACCCGAA
*TIGIT*_forward	TGGTCGCGTTGACTAGAAAGA
*TIGIT*_reverse	GGGCTCCATTCCTCCTGTC
*UQCRFS1*_forward	CGTCACCCAGTTCGTTTCCA
*UQCRFS1*_reverse	AGGGGTTTGCCTCTCCATTTG
*UQCRC1*_forward	GGGGCACAAGTGCTATTGC
*UQCRC1*_reverse	GTTGTCCAGCAGGCTAACC
*CYC1*_forward	CTTCGCGGGGTAGTGTTGG
*CYC1*_reverse	GGCCAGACTTCGACGACAA
*SREBF1*_forward	CGGAACCATCTTGGCAACAGT
*SREBF1*_reverse	CGCTTCTCAATGGCGTTGT
*SREBF2*_forward	AACGGTCATTCACCCAGGTC
*SREBF2*_reverse	GGCTGAAGAATAGGAGTTGCC
*SQLE*_forward	TGACAATTCTCATCTGAGGTCCA

*SQLE*_reverse	CAGGGATACCCTTTAGCAGTTTT
*SCAP*_forward	GTGGACTCTGACCGCAAACAA
*SCAP*_reverse	CGGGACAAAGGTGAACGAAATAC
*SCARB1*_forward	AATAAGCCCATGACCCTGAAGC
*SCARB1*_reverse	GCCCCACATGATCTCACCC
*LDLR*_forward	ACGGCGTCTCTTCCTATGACA
*LDLR*_reverse	CCCTTGGTATCCGCAACAGA
*HMGCR*_forward	TGATTGACCTTTCCAGAGCAAG
*HMGCR*_reverse	CTAAAATTGCCATTCCACGAGC
*HMGCS1*_forward	CATTAGACCGCTGCTATTCTGTC
*HMGCS1*_reverse	TTCAGCAACATCCGAGCTAGA
*PGGT1B*_forward	TCTCCGGGCTGGATATGTTG
*PGGT1B*_reverse	CGGAAACCACAGCGATTTAGAT
*FNTB*_forward	TTTCACCTACTATTGCCCTCCA
*FNTB*_reverse	CGTGACTGTTTCCACCGAGT
*GGPS1*_forward	ACAGCATCTATGGAATCCCATCT
*GGPS1*_reverse	CAAAAGCTGGCGGGTAAAAAG
*PDCD1*_forward	CCAGGATGGTTCTTAGACTCCC
*PDCD1*_reverse	TTTAGCACGAAGCTCTCCGAT
*CD44*_forward	CTGCCGCTTTGCAGGTGTA
*CD44*_reverse	CATTGTGGGCAAGGTGCTATT
*SOAT1*_forward	GAAGTTGGCAGTCACTTTGATGA
*SOAT1*_reverse	GAGCGCACCCACCATTATCTA
*SOAT2*_forward	ATGGAAACACTGAGACGCACA
*SOAT2*_reverse	GGTAGGATTGTATAGCCTCCCG
*CPT1A*_forward	ATCAATCGGACTCTGGAAACGG
*CPT1A*_reverse	TCAGGGAGTAGCGCATGGT
*CCDC58*_forward	AGTGGCGGTGTGAACTGTG
*CCDC58*_reverse	GCTGTTGGAACCGTAGTGTTTA
*LGALS3*_forward	ATGGCAGACAATTTTTCGCTCC
*LGALS3*_reverse	GCCTGTCCAGGATAAGCCC
*STAT5A*_forward	GCAGAGTCCGTGACAGAGG
*STAT5A*_reverse	CCACAGGTAGGGACAGAGTCT
*CREB1*_forward	TTAACCATGACCAATGCAGCA
*CREB1*_reverse	TGGTATGTTTGTACGTCTCCAGA
*CEBPB*_forward	CTTCAGCCCGTACCTGGAG
*CEBPB*_reverse	GGAGAGGAAGTCGTGGTGC
*FASN*_forward	GCAAGCTGAAGGACCTGTCT
*FASN*_reverse	AATCTGGGTTGATGCCTCCG
*ACACA*_forward	ATGTCTGGCTTGCACCTAGTA
*ACACA*_reverse	CCCCAAAGCGAGTAACAAATTCT
*JUN*_forward	TCCAAGTGCCGAAAAAGGAAG
*JUN*_reverse	CGAGTTCTGAGCTTTCAAGGT

*PPARA*_forward	ATGGTGGACACGGAAAGCC
*PPARA*_reverse	CGATGGATTGCGAAATCTCTTGG
*PPARG*_forward	TACTGTCGGTTTCAGAAATGCC
*PPARG*_reverse	GTCAGCGGACTCTGGATTCAG
*NR4A1*_forward	GGCTCGGGGATACTGGATACA
*NR4A1*_reverse	CTGGCATGAAGCGTTGTCC
*NR4A2*_forward	GCACTCCGGGTCGGTTTAC
*NR4A2*_reverse	GCCACGTAGTTCTGGTGGAA
*ECHS1*_forward	TGTCCTGTTGAGACACTGGTG
*ECHS1*_reverse	ACAAACGCGGTCATCCCTTC
*FABP5*_forward	TGAAGGAGCTAGGAGTGGGAA
*FABP5*_reverse	TGCACCATCTGTAAAGTTGCAG
*SDC1*_forward	ACGGCTATTCCCACGTCTC
*SDC1*_reverse	TCTGGCAGGACTACAGCCTC
*MDH1*_forward	TTTGGATCACAACCGAGCTAAAG
*MDH1*_reverse	ACATCTGGATACTGAGTCGAGG
*MDH2*_forward	GCCATGATCTGCGTCATTGC
*MDH2*_reverse	CCGAAGATTTTGTTGGGGTTGT
*ECH1*_forward	TCATCACTCGATACCAGGAGAC
*ECH1*_reverse	GGCACAGTACCGGATGTCA
*ATF4*_forward	CCCTTCACCTTCTTACAACCTC
*ATF4*_reverse	TGCCCAGCTCTAAACTAAAGGA
*RAC2*_forward	CAACGCCTTTCCCGGAGAG
*RAC2*_reverse	TCCGTCTGTGGATAGGAGAGC
*EGR1*_forward	GGTCAGTGGCCTAGTGAGC
*EGR1*_reverse	GTGCCGCTGAGTAAATGGGA
*TBX21*_forward	GTCCAACAATGTGACCCAGAT
*TBX21*_reverse	ACCTCAACGATATGCAGCCG
*MITF*_forward	CAGTCCGAATCGGGGATCG
*MITF*_reverse	TGCTCTTCAGCGGTTGACTTT
*CD36*_forward	CTTTGGCTTAATGAGACTGGGAC
*CD36*_reverse	GCAACAAACATCACCACACCA
*GSTP1*_forward	CCCTACACCGTGGTCTATTTCC
*GSTP1*_reverse	CAGGAGGCTTTGAGTGAGC

Supplementary Table 4 sgRNA sequences used in this study.

sgRNA primers		
Human Gene	Sequencing (5' - 3')	Efficacy score
CD70-sgRNA1_forward	CACCgGGGTTCGGGCTGCTCGGTG	0.77
CD70-sgRNA1_reverse	AAACCACCGAGCAGCCCGAACCCC	
CD70-sgRNA2_forward	CACCgCGGTGCGGCGCAGGCCCTA	0.72
CD70-sgRNA2_reverse	AAACTAGGGCCTGCGCCGCACCGC	
CD70-sgRNA3_forward	CACCgGGTGCGGCGCAGGCCCTAT	0.68
CD70-sgRNA3_reverse	AAACATAGGGCCTGCGCCGCACCC	
CD70-sgRNA4_forward	CACCGGCCCTATGGGTGCGTCCTG	0.74
CD70-sgRNA4_reverse	AAACCAGGACGCACCCATAGGGCC	
CD70-sgRNA5_forward	CACCgGCTTTGGTCCCATTGGTCG	0.74
CD70-sgRNA5_reverse	AAACCGACCAATGGGACCAAAGCC	
Mouse Gene	Sequencing (5' - 3')	Efficacy score
cd70-sgRNA1_forward	CACCgCCAGCAGCAGCCATGGCCAT	0.72
cd70-sgRNA1_reverse	AAACATGGCCATGGCTGCTGCTGGC	
cd70-sgRNA2_forward	CACCGGAAGGTCGCCCTTGCCCCT	0.77
cd70-sgRNA2_reverse	AAACAGGGGCAAGGGCGACCTTCC	
cd70-sgRNA3_forward	CACCgAGGAAGGTCGCCCTTGCCCC	0.66
cd70-sgRNA3_reverse	AAACGGGGCAAGGGCGACCTTCCTC	
cd70-sgRNA4_forward	CACCgTGGCCATTGGCGCTGGAACG	0.73
cd70-sgRNA4_reverse	AAACCGTTCCAGCGCCAATGGCCAC	
cd70-sgRNA5_forward	CACCgTCCCGCTCCAGCGAACCCAG	0.75
cd70-sgRNA5_reverse	AAACCTGGGTTCGCTGGAGCGGGAC	

Supplementary Table 5 Information about antibodies used in this study.

Flow Antibody	Clone number	Vendor	Catalog No.	Dilution	Validation website
PE anti-human FOXP3 Antibody	206D	BioLegend	320108	1:20	https://www.biolegend.com/en-us/products/pe-anti-human-foxp3-antibody-3178?GroupID=BLG8612
APC anti-human CD152 (CTLA-4) Antibody	L3D10	BioLegend	349908	1:20	https://www.biolegend.com/ja-jp/products/apc-anti-human-cd152-ctla-4-antibody-6999
APC anti-human CD27 Antibody	O323	BioLegend	302810	1:50	https://www.biolegend.com/de-at/products/apc-anti-human-cd27-antibody-808?GroupID=BLG7922
APC/Cyanine7 anti-human CD45RO Antibody	UCHL1	BioLegend	304228	1:20	https://www.biolegend.com/en-us/products/apc-cyanine7-anti-human-cd45ro-antibody-7372?GroupID=GROUP658
PE/Cyanine7 anti-human CD25 Antibody	BC96	BioLegend	302612	1:50	https://www.biolegend.com/en-us/products/pe-cyanine7-anti-human-cd25-antibody-1909?GroupID=BLG7919
PE anti-human CD70 Antibody	113-16	BioLegend	355104	1:50	https://www.biolegend.com/en-us/products/pe-anti-human-cd70-antibody-8044?GroupID=BLG10960
FITC anti-human CD45 Antibody	HI30	BioLegend	304038	1:50	https://www.biolegend.com/fr-ch/products/fitc-anti-human-cd45-antibody-707
PE anti-human Granzyme A Antibody	CB9	BioLegend	507206	1:20	https://www.biolegend.com/en-us/products/pe-anti-human-granzyme-a-antibody-1539
PE anti-human Perforin Antibody	B-D48	BioLegend	353304	1:20	https://www.biolegend.com/en-us/products/pe-anti-human-perforin-antibody-7516?GroupID=BLG9616
APC anti-human CD279 (PD-1) Antibody	A17188B	BioLegend	621610	1:20	https://www.biolegend.com/nl-be/products/apc-anti-human-cd279-pd-1-antibody-18920
FITC anti-human CD274 (B7-H1, PD-L1) Antibody	MIH2	BioLegend	393605	1:20	https://www.biolegend.com/en-us/products/fitc-anti-human-cd274-b7-h1-pd-l1-antibody-16037?GroupID=BLG9934

APC anti-human CD70 Antibody	113-16	BioLegend	355110	1:50	https://www.biolegend.com/en-us/products/apc-anti-human-cd70-antibody-8852?GroupID=BLG10960
CD70 Monoclonal Antibody (FR70), PE, eBioscience™	FR70	Invitrogen	12-0701-82	1:20	https://www.thermofisher.com/antibody/product/CD70-Antibody-clone-FR70-Monoclonal/12-0701-82
FITC anti-human/mouse/rat CD278 (ICOS) Antibody	C398.4A	BioLegend	313506	1:20	https://www.biolegend.com/fr-ch/products/fitc-anti-human-mouse-rat-cd278-icos-antibody-2481
PE/Cyanine7 anti-human CD357 (GITR) Antibody	108-17	BioLegend	371223	1:50	https://www.biolegend.com/nl-be/search-results/pe-cyanine7-anti-human-cd357-gitr-antibody-14456
Pacific Blue™ anti-human FOXP3 Antibody	206D	BioLegend	320116	1:20	https://www.biolegend.com/en-us/products/pacific-blue-anti-human-foxp3-antibody-3053?GroupID=BLG4131
FITC anti-human CD64 Antibody	S18012C	BioLegend	399506	1:50	https://www.biolegend.com/nl-nl/products/fitc-anti-human-cd64-antibody-19189?GroupID=GROUP28
APC anti-human CD138 (Syndecan-1) Antibody	DL-101	BioLegend	352308	1:50	https://www.biolegend.com/en-us/search-results/apc-anti-human-cd138-syndecan-1-antibody-7315
PE anti-human CD45RA Antibody	HI100	BioLegend	304107	1:50	https://www.biolegend.com/en-us/products/pe-anti-human-cd45ra-antibody-687
PE anti-human CD366 (Tim-3) Antibody	F38-2E2	BioLegend	345006	1:20	https://www.biolegend.com/en-us/products/pe-anti-human-cd366-tim-3-antibody-6121?GroupID=BLG9937
FITC anti-human CD4 Antibody	A161A1	BioLegend	357406	1:50	https://www.biolegend.com/en-gb/products/fitc-anti-human-cd4-antibody-8738?GroupID=BLG11451
FITC anti-human CD8a Antibody	RPA-T8	BioLegend	301006	1:50	https://www.biolegend.com/fr-lu/products/fitc-anti-human-cd8a-antibody-834
APC anti-human CD8 Antibody	SK1	BioLegend	344722	1:50	https://www.biolegend.com/en-us/products/apc-anti-human-cd8-antibody-6531?GroupID=BLG10167
IHC/IF Antibody	Clone	Vendor	Catalog No.	Dillution	

CD70 Polyclonal Antibody	Polyclonal	Invitrogen	PA5-102557	1:200	https://www.thermofisher.com/antibody/product/CD70-Antibody-Polyclonal/PA5-102557
Anti-FOXP3 Antibody	mAbcam 22510	Abcam	ab22510	1:100	https://www.abcam.com/products/primary-antibodies/foxp3-antibody-mabcam-22510-bsa-and-azide-free-ab188638.html
Recombinant anti-CTLA4 Antibody	CAL49	Abcam	ab237712	1:500	https://www.abcam.com/products/primary-antibodies/ctla4-antibody-cal49-ab237712.html
Goat anti-Mouse IgG (H+L) Cross-Adsorbed Secondary Antibody, Alexa Fluor™ 488	Polyclonal	Invitrogen	A-11001	1:2000	https://www.thermofisher.com/antibody/product/Goat-anti-Mouse-IgG-H-L-Cross-Adsorbed-Secondary-Antibody-Polyclonal/A-11001
Donkey anti-Rabbit IgG (H+L) Highly Cross-Adsorbed Secondary Antibody, Alexa Fluor™ 555	Polyclonal	Invitrogen	A-31572	1:1000	https://www.thermofisher.com/antibody/product/Donkey-anti-Rabbit-IgG-H-L-Highly-Cross-Adsorbed-Secondary-Antibody-Polyclonal/A-31572

Supplementary Table 6 shRNA sequences used in this study.

shRNA primers	
Gene	Sequencing (5' - 3')
NFKB2-shRNA_forward	CcggGCTGCTAAATGCTGCTCAGAACTCGAGTTCTGAGCAGCATTTAGCAGCTTTTTg
NFKB2-shRNA_reverse	aattcaaaaaGCTGCTAAATGCTGCTCAGAACTCGAGTTCTGAGCAGCATTTAGCAGC

References

1. Liu, D., *et al.* Integrative molecular and clinical modeling of clinical outcomes to PD1 blockade in patients with metastatic melanoma. *Nat Med* **25**, 1916-1927 (2019).
2. Van Allen, E.M., *et al.* Genomic correlates of response to CTLA-4 blockade in metastatic melanoma. *Science* **350**, 207-211 (2015).
3. Sade-Feldman, M., *et al.* Defining T Cell States Associated with Response to Checkpoint Immunotherapy in Melanoma. *Cell* **175**, 998-1013 e1020 (2018).
