# Supplementary material for: Nasopharyngeal carcinoma cells promote regulatory T cell development and suppressive activity via CD70-CD27 interaction
Source: Nat Commun. 2023 Apr 6;14:1912. doi: 10.1038/s41467-023-37614-6 (PMC10079957; doi:10.1038/s41467-023-37614-6)
Supplement: Supplementary file 3 — Description of Additional Supplementary Files [file 41467_2023_37614_MOESM3_ESM.pdf]

Description of Additional Supplementary Files

Supplementary Data 1: Differentially-expressed genes in Treg subtypes.

Supplementary Data 2: Transcription factor activities in NPC and NPE cells.

Supplementary Data 3: Mass spectrometry data
