# Supplementary material for: Nasopharyngeal carcinoma cells promote regulatory T cell development and suppressive activity via CD70-CD27 interaction
Source: Nat Commun. 2023 Apr 6;14:1912. doi: 10.1038/s41467-023-37614-6 (PMC10079957; doi:10.1038/s41467-023-37614-6)
Supplement: Supplementary file 7 — Reporting Summary [file 41467_2023_37614_MOESM7_ESM.pdf]

nature portfolio

Corresponding author(s): Xin-Yuan Guan

Last updated by author(s): Mar 7, 2023

Reporting Summary

Nature Portfolio wishes to improve the reproducibility of the work that we publish. This form provides structure for consistency and transparency in reporting. For further information on Nature Portfolio policies, see our Editorial Policies and the Editorial Policy Checklist.

Statistics

For all statistical analyses, confirm that the following items are present in the figure legend, table legend, main text, or Methods section.

n/a	Confirmed	
☐	☒	The exact sample size (*n*) for each experimental group/condition, given as a discrete number and unit of measurement
☐	☒	A statement on whether measurements were taken from distinct samples or whether the same sample was measured repeatedly
☐	☒	The statistical test(s) used AND whether they are one- or two-sided<br>*Only common tests should be described solely by name; describe more complex techniques in the Methods section.*
☐	☒	A description of all covariates tested
☐	☒	A description of any assumptions or corrections, such as tests of normality and adjustment for multiple comparisons
☐	☒	A full description of the statistical parameters including central tendency (e.g. means) or other basic estimates (e.g. regression coefficient) AND variation (e.g. standard deviation) or associated estimates of uncertainty (e.g. confidence intervals)
☐	☒	For null hypothesis testing, the test statistic (e.g. *F*, *t*, *r*) with confidence intervals, effect sizes, degrees of freedom and *P* value noted<br>*Give P values as exact values whenever suitable.*
☒	☐	For Bayesian analysis, information on the choice of priors and Markov chain Monte Carlo settings
☒	☐	For hierarchical and complex designs, identification of the appropriate level for tests and full reporting of outcomes
☐	☒	Estimates of effect sizes (e.g. Cohen's *d*, Pearson's *r*), indicating how they were calculated

Our web collection on statistics for biologists contains articles on many of the points above.

Software and code

Policy information about availability of computer code

Data collection	Softwares used for transcriptome data collection, quality control and aggregation: R v 4.2.2, R studio v 2022.12.0+353, Python v 3.6, Cell Ranger v 6.1.2, and Space Ranger v 1.3.1. For flow cytometry data collection and analysis: NovoExpress software (v 1.5.6, Agilent). For mass spectrometry analysis: Agilent MassHunter Workstation Quantitative Analysis Software (v B.04.xx, Agilent).
Data analysis	For single-cell data aggregation, the raw fastq data of 50 samples from Gong et al., Liu et al., and Chen et al. NPC single cell cohorts were processed and aggregated by Cell Ranger (v 6.1.2, 10x Genomics). Batch effects among single cells originated from different studies were corrected by Harmony (v 1.0, Broad Institute).<br>For the aggregated single-cell data, the gene expression matrix metadata were converted to a Seurat object using Seurat R package (v 4.0). Single cells with unique feature counts >4000 or <200, or >15% mitochondrial counts, were filtered out from the downstream analysis. Doublets were removed by DoubletFinder (v 2.0). UMAP reduction was performed to identify cell clusters.<br>For processing the bulk RNA-seq data, the read counts in each RNA-seq cohort were generated by HTSeq (version 0.9.1) and normalized by DESeq2 (version 1.22.2). The quality of RNA-seq data was evaluated by Picard metrics (version 2.17.4) and RSeQC (version 2.6.4).<br>The spatial transcriptome library was sequenced on a NovaSeq 6000 (Illumina) and mapped to the human genome (hg38) using Spatial Ranger (v 1.3.1, 10× Genomics). The spatial sequencing data were normalized using the SCTransform function followed by PCA reduction. Since each spatial spot contained multiple cells with varied lineages, we deconvoluted the spatial data using the SCTransformed NPC scRNA-seq data as a reference and estimated the underlying cellular composition in each voxel via the FindTransferAnchors function (normalization method = "SCT") and the TransferData function (anchorset = anchors identified by the FindTransferAnchors function), implemented in Seurat (v 4.0).

For manuscripts utilizing custom algorithms or software that are central to the research but not yet described in published literature, software must be made available to editors and reviewers. We strongly encourage code deposition in a community repository (e.g. GitHub). See the Nature Portfolio guidelines for submitting code & software for further information.

Data

Policy information about availability of data

All manuscripts must include a data availability statement. This statement should provide the following information, where applicable:

- Accession codes, unique identifiers, or web links for publicly available datasets
- A description of any restrictions on data availability
- For clinical datasets or third party data, please ensure that the statement adheres to our policy

The NPC single-cell sequencing data used in the study are publicly available in Gene Expression Omnibus (GEO) under accession numbers GSE150825 [https://www.ncbi.nlm.nih.gov/geo/query/acc.cgi?acc=GSE150825], GSE150430 [https://www.ncbi.nlm.nih.gov/geo/query/acc.cgi?acc=GSE150430], and GSE162025 [https://www.ncbi.nlm.nih.gov/geo/query/acc.cgi?acc=GSE162025]. The NPC bulk RNA sequencing data used in the study are publicly available in GEO under accession numbers GSE68799 [https://www.ncbi.nlm.nih.gov/geo/query/acc.cgi?acc=GSE68799], GSE102349 [https://www.ncbi.nlm.nih.gov/geo/query/acc.cgi?acc=GSE102349], GSE53819 [https://www.ncbi.nlm.nih.gov/geo/query/acc.cgi?acc=GSE53819], GSE13597 [https://www.ncbi.nlm.nih.gov/geo/query/acc.cgi?acc=GSE13597], and GSE118719 [https://www.ncbi.nlm.nih.gov/geo/query/acc.cgi?acc=GSE118719]. The RNA sequencing data for time-resolved induced Treg differentiation used in the study is publicly available in GEO under accession number GSE96538 [https://www.ncbi.nlm.nih.gov/geo/query/acc.cgi?acc=GSE96538]. The HNSCC single-cell sequencing data used in the study are publicly available in GEO under accession numbers GSE139324 [https://www.ncbi.nlm.nih.gov/geo/query/acc.cgi?acc=GSE139324] and GSE164690 [https://www.ncbi.nlm.nih.gov/geo/query/acc.cgi?acc=GSE164690]. The raw and processed Visium spatial sequencing data of primary NPC tissues and single-cell sequencing data of the co-cultured PBMCs have been deposited in GEO under accession numbers GSE200310 [https://www.ncbi.nlm.nih.gov/geo/query/acc.cgi?acc=GSE200310] and GSE200315 [https://www.ncbi.nlm.nih.gov/geo/query/acc.cgi?acc=GSE200315]. Source data are provided with this paper. The remaining data are available within the Article, Supplementary Information or Source Data file, or are available from the corresponding author upon reasonable request.

Human research participants

Policy information about studies involving human research participants and Sex and Gender in Research.

Reporting on sex and gender	There is no sex and gender requirements and bias in this study.
Population characteristics	Male and female patients with primary NPC and healthy blood donors with the age ranging from 21 to 63 years old were enrolled in this study.
Recruitment	For experiments and sequencing using primary NPC tissues and blood samples, we obtained these biological samples from NPC patients diagnosed at the Queen Mary Hospital and The University of Hong Kong-Shenzhen Hospital. For experiments using human PBMCs, we obtained and isolated the whole blood from healthy blood donors at the Hong Kong Red Cross. There are no specific requirements and self-selection biases for NPC patient and healthy blood donor recruitment in this study.
Ethics oversight	The study was approved by the ethics committee at the University of Hong Kong, the University of Hong Kong-Shenzhen Hospital and the Hong Kong Red Cross. We complied with all related ethical regulations. Written informed consent was obtained from healthy blood donors and all patients with primary NPC and non-malignant nasopharyngeal inflammation for their tissues to be used in the spatial transcriptome sequencing, IHC/IF staining, flow cytometry analysis and primary culture in this study.

Note that full information on the approval of the study protocol must also be provided in the manuscript.

Field-specific reporting

Please select the one below that is the best fit for your research. If you are not sure, read the appropriate sections before making your selection.

[x] Life sciences   [ ] Behavioural & social sciences   [ ] Ecological, evolutionary & environmental sciences

For a reference copy of the document with all sections, see nature.com/documents/nr-reporting-summary-flat.pdf

Life sciences study design

All studies must disclose on these points even when the disclosure is negative.

Sample size	For our single-cell and bulk RNA-seq analysis, no sample size calculation was performed because the data are collected from publicly available cohorts with predefined sample sizes which were determined by sample availability in each study (PMID: 33750785, 33531485, 32686767, 28851814 and 30477559). For the Visium spatial sequencing and all experiments using primary NPC tissues and blood samples, no sample size calculation was performed either because the total number of tissues and blood samples that we have collected are based on patient/donor availability at our hospitals and Hong Kong Red Cross. For other in vitro and in vivo experiments in this study, no prior sample size calculation was performed. We chose to perform at least three independent experiments in order to sufficiently compute statistical significance.

Data exclusions	Sequenced samples that fail to meet the quality control requirements stated above are excluded from downstream analysis. Cells in our integrated NPC single-cell cohort that are determined to be empty droplets, doublets and dead cells are excluded from the downstream analysis.
Replication	All in vitro and in vivo experiments were independently performed at least three times to compute statistical significance. And all attempts at replication were successful for all in vitro and in vivo experiments. For computational results using publicly available data, the replication is not relevant since we evaluated statistical significance based on all available samples.
Randomization	Randomization was performed on tumor-bearing humanized mice who received control, anti-CD70, anti-PD-1 and anti-CD70+anti-PD-1 treatments. The tumor-bearing mice were ear-tagged and randomly distributed by shuffling their identities initially into different treatment groups.
Blinding	For experiments using humanized mice and NPC organoids, the tumor size on each mouse and the number of organoids in each well were determined in a double-blinded manner by a person who was not the one who performed the experiments. For other experiments, blinding was not performed because we analyzed the results objectively by using equipment instead of by humans.

Reporting for specific materials, systems and methods

We require information from authors about some types of materials, experimental systems and methods used in many studies. Here, indicate whether each material, system or method listed is relevant to your study. If you are not sure if a list item applies to your research, read the appropriate section before selecting a response.

Materials & experimental systems

n/a	Involved in the study
☐	☒ Antibodies
☐	☒ Eukaryotic cell lines
☒	☐ Palaeontology and archaeology
☐	☒ Animals and other organisms
☒	☐ Clinical data
☒	☐ Dual use research of concern

Methods

n/a	Involved in the study
☒	☐ ChIP-seq
☐	☒ Flow cytometry
☒	☐ MRI-based neuroimaging

Antibodies

Antibodies used

Flow Antibody,Clone, Vendor, Catalog No., Dillution
PE anti-human FOXP3 Antibody, 206D, BioLegend, 320108, 1:20
APC anti-human CD152 (CTLA-4) Antibody, L3D10, BioLegend, 349908, 1:20
APC anti-human CD27 Antibody, O323, BioLegend, 302810, 1:50
APC/Cyanine7 anti-human CD45RO Antibody, UCHL1, BioLegend, 304228, 1:20
PE/Cyanine7 anti-human CD25 Antibody, BC96, BioLegend, 302612, 1:50
PE anti-human CD70 Antibody, 113-16, BioLegend, 355104, 1:20
FITC anti-human CD45 Antibody, HI30, BioLegend, 304038, 1:50
PE anti-human Granzyme A Antibody, CB9, BioLegend, 507206, 1:20
PE anti-human Perforin Antibody, B-D48, BioLegend, 353304, 1:20
APC anti-human CD279 (PD-1) Antibody, A17188B, BioLegend, 621610, 1:20
FITC anti-human CD274 (B7-H1, PD-L1) Antibody, MIH2, BioLegend, 393605, 1:20
APC anti-human CD70 Antibody 113-16, BioLegend, 355110, 1:50
CD70 Monoclonal Antibody (FR70), PE, eBioscience™, FR70, Invitrogen, 12-0701-82, 1:20
FITC anti-human/mouse/rat CD278 (ICOS) Antibody, C398.4A, BioLegend, 313506, 1:20
PE/Cyanine7 anti-human CD357 (GITR) Antibody, 108-17, BioLegend, 371223, 1:50
Pacific Blue™ anti-human FOXP3 Antibody, 206D, BioLegend, 320116, 1:20
FITC anti-human CD64 Antibody, S18012C, BioLegend, 399506, 1:50
APC anti-human CD138 (Syndecan-1) Antibody, DL-101, BioLegend, 352308, 1:50
PE anti-human CD45RA Antibody, HI100, BioLegend, 304107, 1:50
PE anti-human CD366 (Tim-3) Antibody, F38-2E2, BioLegend, 345006, 1:20
FITC anti-human CD4 Antibody, A161A1, BioLegend, 357406, 1:50
FITC anti-human CD8a Antibody, RPA-T8, BioLegend, 301006, 1:50
APC anti-human CD8 Antibody, SK1, BioLegend, 344722, 1:50

IHC/IF Antibody, Clone, Vendor, Catalog No., Dillution
CD70 Polyclonal Antibody Polyclonal, Invitrogen, PA5-102557, 1:200
Anti-FOXP3 Antibody, mAbcam 22510, Abcam, ab22510, 1:100
Recombinant anti-CTLA4 Antibody, CAL49, Abcam, ab237712, 1:500
Goat anti-Mouse IgG (H+L) Cross-Adsorbed Secondary Antibody, Alexa Fluor™ 488, Polyclonal, Invitrogen, A-11001, 1:2000
Donkey anti-Rabbit IgG (H+L) Highly Cross-Adsorbed Secondary Antibody, Alexa Fluor™ 555, Polyclonal, Invitrogen, A-31572, 1:1000

Validation	The antibodies for flow cytometry analysis, IHC and IF staining were commercially available from BioLegend, Abcam and Invitrogen. Validation of these antibodies has been performed by respective manufactures and our routine laboratory work. Additional validation information is provided in Supplementary Table 5.<br><br>PE anti-human FOXP3 Antibody https://www.biolegend.com/en-us/products/pe-anti-human-foxp3-antibody-3178?GroupID=BLG8612<br>APC anti-human CD152 (CTLA-4) Antibody https://www.biolegend.com/ja-jp/products/apc-anti-human-cd152-ctla-4-antibody-6999<br>APC anti-human CD27 Antibody https://www.biolegend.com/de-at/products/apc-anti-human-cd27-antibody-808?GroupID=BLG7922<br>APC/Cyanine7 anti-human CD45RO Antibody https://www.biolegend.com/en-us/products/apc-cyanine7-anti-human-cd45ro-antibody-7372?GroupID=GROUP658<br>PE/Cyanine7 anti-human CD25 Antibody https://www.biolegend.com/en-us/products/pe-cyanine7-anti-human-cd25-antibody-1909?GroupID=BLG7919<br>PE anti-human CD70 Antibody https://www.biolegend.com/en-us/products/pe-anti-human-cd70-antibody-8044?GroupID=BLG10960<br>FITC anti-human CD45 Antibody https://www.biolegend.com/fr-ch/products/fitc-anti-human-cd45-antibody-707<br>PE anti-human Granzyme A Antibody https://www.biolegend.com/en-us/products/pe-anti-human-granzyme-a-antibody-1539<br>PE anti-human Perforin Antibody https://www.biolegend.com/en-us/products/pe-anti-human-perforin-antibody-7516?GroupID=BLG9616<br>APC anti-human CD279 (PD-1) Antibody https://www.biolegend.com/nl-be/products/apc-anti-human-cd279-pd-1-antibody-18920<br>FITC anti-human CD274 (B7-H1, PD-L1) Antibody https://www.biolegend.com/en-us/products/fitc-anti-human-cd274-b7-h1-pd-l1-antibody-16037?GroupID=BLG9934<br>APC anti-human CD70 Antibody https://www.biolegend.com/en-us/products/apc-anti-human-cd70-antibody-8852?GroupID=BLG10960<br>CD70 Monoclonal Antibody (FR70), PE, eBioscience™ https://www.thermofisher.com/antibody/product/CD70-Antibody-clone-FR70-Monoclonal/12-0701-82<br>FITC anti-human/mouse/rat CD278 (ICOS) Antibody https://www.biolegend.com/fr-ch/products/fitc-anti-human-mouse-rat-cd278-icos-antibody-2481<br>PE/Cyanine7 anti-human CD357 (GITR) Antibody https://www.biolegend.com/nl-be/search-results/pe-cyanine7-anti-human-cd357-gitr-antibody-14456<br>Pacific Blue™ anti-human FOXP3 Antibody https://www.biolegend.com/en-us/products/pacific-blue-anti-human-foxp3-antibody-3053?GroupID=BLG4131<br>FITC anti-human CD64 Antibody https://www.biolegend.com/nl-nl/products/fitc-anti-human-cd64-antibody-19189?GroupID=GROUP28<br>APC anti-human CD138 (Syndecan-1) Antibody https://www.biolegend.com/en-us/search-results/apc-anti-human-cd138-syndecan-1-antibody-7315<br>PE anti-human CD45RA Antibody https://www.biolegend.com/en-us/products/pe-anti-human-cd45ra-antibody-687<br>PE anti-human CD366 (Tim-3) Antibody https://www.biolegend.com/en-us/products/pe-anti-human-cd366-tim-3-antibody-6121?GroupID=BLG9937<br>FITC anti-human CD4 Antibody https://www.biolegend.com/en-gb/products/fitc-anti-human-cd4-antibody-8738?GroupID=BLG11451<br>FITC anti-human CD8a Antibody https://www.biolegend.com/fr-lu/products/fitc-anti-human-cd8a-antibody-834<br>APC anti-human CD8 Antibody https://www.biolegend.com/en-us/products/apc-anti-human-cd8-antibody-6531?GroupID=BLG10167<br>CD70 Polyclonal Antibody https://www.thermofisher.com/antibody/product/CD70-Antibody-Polyclonal/PA5-102557<br>Anti-FOXP3 Antibody https://www.abcam.com/products/primary-antibodies/foxp3-antibody-mabcam-22510-bsa-and-azide-free-ab188638.html<br>Recombinant anti-CTLA4 Antibody https://www.abcam.com/products/primary-antibodies/ctla4-antibody-cal49-ab237712.html<br>Goat anti-Mouse IgG (H+L) Cross-Adsorbed Secondary Antibody, Alexa Fluor™ 488 https://www.thermofisher.com/antibody/product/Goat-anti-Mouse-IgG-H-L-Cross-Adsorbed-Secondary-Antibody-Polyclonal/A-11001<br>Donkey anti-Rabbit IgG (H+L) Highly Cross-Adsorbed Secondary Antibody, Alexa Fluor™ 555 https://www.thermofisher.com/antibody/product/Donkey-anti-Rabbit-IgG-H-L-Highly-Cross-Adsorbed-Secondary-Antibody-Polyclonal/A-31572

Eukaryotic cell lines

Policy information about cell lines and Sex and Gender in Research

Cell line source(s)	C666, NP460, NP69, NPC43-EBV-negative, NPC43-EBV-postive cell lines were provided by Prof. George Sai Wah Tsao whose lab had previously established these immortalized NPC and normal nasopharyngeal epithelial cell lines. The 293FT cell line were purchased from Invitrogen (R70007).
Authentication	All cell lines are tested to be authentic by using STR profiling and free of cross-contamination.
Mycoplasma contamination	All cell lines used in the study were tested negative for mycoplasma contamination.
Commonly misidentified lines (See ICLAC register)	No commonly misidentified lines were used in the study.

Animals and other research organisms

Policy information about studies involving animals; ARRIVE guidelines recommended for reporting animal research, and Sex and Gender in Research

Laboratory animals	NOD.Cg-PrkdcscidIl2rgtm1Wjl/SzJ (NSG) mice and C57BL/6J mice were purchased from the Centre for Comparative Medicine Research, the University of Hong Kong. Female NSG mice at 6 weeks of age were used for PBMC-engrafted immune humanization. Male or female C57BL/6J mice at 4-6 weeks of age were used for melanoma inoculation. The maximal end-point tumor size permitted by the ethics committee is 2 cm in diameter and no tumor burden exceeded this limit in our study. Mice were housed on 12-hour light-dark cycles with the ambient temperature at 20-25°C and 40-60% humidity at the Centre for Comparative Medicine Research, the University of Hong Kong.
Wild animals	No wild animals were used in the study.
Reporting on sex	There is no sex bias in this study. Female mice were chosen for immune humanization because they were more manageable compared to male ones.
Field-collected samples	No field collected samples were used in the study.
Ethics oversight	All animal experiments were approved by the Committee of the Use of Live Animals in Teaching and Research at the University of Hong Kong under protocol 4924-19 and the ethics committee at the University of Hong Kong.

Note that full information on the approval of the study protocol must also be provided in the manuscript.

Flow Cytometry

Plots

Confirm that:

- [x] The axis labels state the marker and fluorochrome used (e.g. CD4-FITC).
- [x] The axis scales are clearly visible. Include numbers along axes only for bottom left plot of group (a 'group' is an analysis of identical markers).
- [x] All plots are contour plots with outliers or pseudocolor plots.
- [x] A numerical value for number of cells or percentage (with statistics) is provided.

Methodology

Sample preparation	For surface marker staining, cells were first washed by cell staining buffer (Biolegend) twice. Targeted antibodies were diluted in cell staining buffer as based on the above-stated dilution factor. Cells were suspended in antibody-containing cell staining buffer with a concentration <1 million cells/100 µL, and incubated at 4 °C for 15 min. Subsequently, 1 mL of cell staining buffer was added in the cell mixture and centrifuged at 970 rpm (tumor cells) or 300 g (immune cells) for 3 min/5 mins. Cells were washed by 1 mL cell staining buffer again and then suspended in 500 µL cell staining buffer for flow cytometry analysis.<br><br>For intracellular staining, cells were first washed by cell staining buffer (Biolegend) twice. Subsequently, cells were fixed and permeabilized using the Cyto-Fast™ Fix/Perm Buffer Set (Biolegend), as per manufacture protocol. Targeted antibodies were diluted in cell staining buffer as based on the above-stated dilution factor. Cells were suspended in antibody-containing Perm Wash solution with a concentration <1 million cells/100 µL, and incubated at room temperature for 30 min. Subsequently, 2 mL of 1x Perm Wash solution was added in the cell mixture and centrifuged at 300 g for 5 mins. Cells were washed by 1 mL cell staining buffer again and then suspended in 500 µL cell staining buffer for flow cytometry analysis.<br><br>For transcription factor staining, cells were first washed by cell staining buffer (Biolegend) twice. Subsequently, cells were fixed and permeabilized using the True-Nuclear Transcription Factor Buffer Set (Biolegend), as per manufacture protocol. Targeted antibodies were diluted in 1x Perm buffer as based on the above-stated dilution factor. Cells were suspended in antibody-containing cell staining buffer with a concentration <1 million cells/100 µL, and incubated at room temperature for 30 min. Subsequently, 2 mL of 1x Perm buffer was added in the cell mixture and centrifuged at 300 g for 5 mins. Cells were washed by 1 mL cell staining buffer again and then suspended in 500 µL cell staining buffer for flow cytometry analysis.
Instrument	Agilent Novocyte Advanteon BVR, Agilent Novocyte Advanteon BVYG, Agilent NovoCyte Quanteon, BD FACSAria SORP and BD FACSMelody
Software	NovoExpress software (v 1.5.6, Agilent)
Cell population abundance	The positive fractions and median intensities among cell populations were shown and compared within each figure. The post-sort fraction of CD70- cells in C666 and B16-F10 cells changed from 50% (the CD70- fraction in C666 cells after initial CD70-KO) and 70% (B16-F10) to 100%. The purity of sorted samples were determined by staining PE anti-human or anti-mouse antibodies.
Gating strategy	We used the forward scatter area and height (FSC-A and FSC-H), and side scatter area and height (SSC-A or SSC-H) for dead cells/debris removal and single-cell gating. We also used CD45, CD4 and CD8 antibodies to gate targeted CD45- tumor/CD4+

T /CD8+ T subpopulations in our co-culture systems.

- [x] Tick this box to confirm that a figure exemplifying the gating strategy is provided in the Supplementary Information.
